# Recycle of Immobilized Endocellulases in Different Conditions for Cellulose Hydrolysis

**DOI:** 10.1155/2017/4362704

**Published:** 2017-03-29

**Authors:** D. F. Silva, A. F. A. Carvalho, T. Y. Shinya, G. S. Mazali, R. D. Herculano, P. Oliva-Neto

**Affiliations:** ^1^Biological Science Department, Universidade Estadual Paulista (UNESP), Avenida Dom Antônio, 2100 Bairro, Parque Universitário, 19806-900 Assis, SP, Brazil; ^2^Bioprocess & Biotechnology Department, Universidade Estadual Paulista (UNESP), Rod. Araraquara-Jaú Km 1 Bairro, Machados, 14800-901 Araraquara, SP, Brazil

## Abstract

The immobilization of cellulases could be an economical alternative for cost reduction of enzyme application. The derivatives obtained in the immobilization derivatives were evaluated in recycles of paper filter hydrolysis. The immobilization process showed that the enzyme recycles were influenced by the shape (drop or sheet) and type of the mixture. The enzyme was recycled 28 times for sheets E′ and 13 times for drops B′. The derivative E′ showed the highest stability in the recycle obtaining 0.05 FPU/g, RA of 10%, and FPU Yield of 1.64 times, higher than FPU spent or Net FPU Yield of 5.3 times, saving more active enzymes. The derivative B showed stability in recycles reaching 0.15 FPU/g of derivative, yield of Recovered Activity (RA) of 25%, and FPU Yield of 1.57 times, higher than FPU spent on immobilization or Net PFU Yield of 2.81 times. The latex increased stability and resistance of the drops but did not improve the FPU/gram of derivative.

## 1. Introduction

The possible use of renewable fuels has aroused an increasing interest all over the world [1]. This fact has happened due to the positive impacts of replacing fossil fuels with renewable energy. Biofuels are renewable, available, and ecologically friendly [1]. As already reported by Vásquez et al. [2], new studies are being done to develop biotechnological processes that allow the use of lignocellulosic biomass waste, like corn and rice straw and bagasse from sugar cane and pulp industry waste, among others abundantly produced in the world. These residues will be used for the production of biofuels such as second-generation bioethanol. The production of sugarcane in 2016/17 will be increased by 2.9% in relation to the previous season. In absolute numbers, production of 684.77 million tons of sugar cane is estimated, compared to 665.59 million tons in 2015/16 [3]. Considering also that the sugar and alcohol industries generate 135 kg of dried bagasse per ton of crushed cane [4], the total bagasse generated in this season was about 80.5 billion tons.

In order to carry out the hydrolysis of these residues, it is necessary to use cellulases, which are usually produced by filamentous fungi [5]. This hydrolysis is made by reducing the biomass into mainly glucose and xylose, which in turn can be fermented by facultative microorganisms such as* Saccharomyces cerevisiae*, and then converted into bioethanol [2, 5].

The enzymatic conversion of cellulose to glucose is difficult due to the physical nature of the substrate, which is composed mainly of insoluble crystalline fibers (microfibrils) in which the hydrogen bonds hold the molecules together. These fibers are embedded in a matrix of hemicellulose and lignin [6] decreasing the accessibility of cellulolytic enzymes (Beguin, 1990). The cellulases produced by fungi have three main components: endoglucanases that hydrolyze internal *β*1,4 D-glycosidic bonds; the cellobiohydrolases (exocellulase) which produce cellobiose from nonreducing ends from cellulose; the *β*-glucosidases (cellobiases) which convert cellobiose to glucose. For effective hydrolysis of cellulose a consortium of these enzymes in a synergistic action are required (Lynd et al., 2002) [7].

However, the high cost of these enzymes has limited the economic viability of their use in industrial bioprocesses [8]. Therefore, to make them economically feasible, the reuse of immobilized enzyme may be an alternative [9, 10].

Alginate is a natural polysaccharide widely used as support in immobilization by microencapsulation technologies and composed of alternating chains of *α*-L-guluronic acid and *β*-D-mannuronic acid residues [11, 12]. Alginate supports are usually made by cross-linking the carboxyl group of the *α*-L-guluronic acid with a cationic gelling solution such as calcium chloride or barium chloride [13], mixed or not with the solution containing the biocatalyst, depending on the derivative of immobilization [14].

Chitosan is a cationic biopolymer obtained by deacetylation of chitin. This polymer has two functional groups, amino and hydroxyl residues, being used as sites of reaction and coordination. This polysaccharide comprises a linear sequence of sugar monomers *β*-(1-4) 2-acetamide-2-deoxy-D-glucose (N-acetylglucosamine) bases [10, 15, 16]. Natural rubber latex (NRL, cis-1,4-polyisoprene) extracted from* Hevea brasiliensis* has been widely used as a raw material in the manufacturing of gloves, condoms, balloons, and other medical and dental devices. However, recently several new biomedical applications have been proposed using a different manufacturing process [17, 18]. Therefore, due to the porosity of the membrane, ease in handling, low cost, and the possibility of numerous modifications, the latex can be an excellent support for enzyme immobilization.

Hybrid chitosan-alginate is reported in the literature as a support for enzyme immobilization [19]. Chitosan is a polycationic polymer and alginate is a polyanionic polymer, so ionic interactions between them allow rigid gels to form [20]. Busto et al. [21] immobilized microbial endo-*β*-glucanase in alginate beads retaining 75% of its original activity. Wu et al. [22] immobilized cellulase in nanofibrous of PVA membranes by electrospinning retaining 36% of initial activity after six cycles of reuse. Chang et al. [23] used mesoporous silica nanoparticles (MSNs) in cellulase immobilization for conversion of cellulose into glucose. Cellulase chemically linked to MSNs exhibited a large pore size which was responsible for effective cellulose-to-glucose conversion exceeding 80% yield and excellent stability. Romo-Sánchez et al. [24] working with cellulases and xylanases immobilization performed 19 cycles maintaining 64% of the enzymatic activity. Zhang et al. [25] immobilized cellulases on modified silica gel and obtained significant activity over multiple reuses, with 82% and 31% of activity after 7 and 15 recycles, respectively. Song et al. [26] using super paramagnetic nanoparticles immobilized cellulases and reported 85% and 43% of the initial immobilized enzyme activities after being recycled 3 and 10 times, respectively.

This work studies the immobilization and recycling of cellulases, aiming at future applications in the production of second-generation ethanol.

## 2. Materials and Methods

### 2.1. Reagents, Supports, and Enzyme Used in Immobilization Process

The supports used in immobilization process of cellulases were chitosan (C_12_H_24_N_2_O_9_) high molecular weight (Aldrich® code 419419-50 G); sodium alginate (Labsynth® code A1089.01.AF); zeolite (mx/n [(AlO_2_) × (SiO_2_) y]·wH_2_O) (Sigma); cationic and anionic exchanger resin (Amberlite® MB-20, Dow Chemical Comp., US) and polystyrene (Styrofoam®); natural latex (extracted in the farm of ESALQ-USP, Piracicaba, SP); 25% (m/m) glutaraldehyde (Nuclear, Brazil); calcium chloride (Vetec) and acetate buffer (Impex, pH 5.6). As substrate for determination of hydrolytic activity, Filter Paper Whatman no. 1 was used in the technique of FPase. The enzyme cellulase (EC3.2.1.4, 1,4-*β*-endoglucanase, ROHAMENT®CL, AB Enzymes, Darmstadt, Germany) from* Trichoderma reesei* was used for immobilization.

### 2.2. Maintenance of Latex

The latex was extracted at BDF Rubber Latex Co. Ltd (producer and distributor of concentrated rubber latex), Guarantã, Brazil. The latex solution extracted from* Hevea brasiliensis* consisted of a mixture of different clones. After extraction, ammonia was used to keep the latex liquid, and this material was centrifuged at 8000 rpm. The centrifugation was important since this process decreased some proteins contained in natural latex that cause allergic reactions [17].

### 2.3. The Immobilization Process

#### 2.3.1. 1,4-*β*-Endoglucanase Immobilization on Activated Carbon, Zeolite, Ion Exchange Resin, and Polystyrene

Preliminary tests with some supports (activated carbon, zeolite, ion exchange resin, and polystyrene) were conducted for cellulase immobilization to determine which would be more efficient in the process of enzyme immobilization in sheets (derivatives E, E′, J, and J′, Table 1). Polystyrene was previously treated with the following procedure: autoclavation for 15 min at 120°C, and subsequently maintenance in 1 : 2 (w·v^−1^) 50% (v·v^−1^) ethanol solution for 30 min at 28°C. Then this support was washed with deionized water (1000 mL), modified derivative by Hou et al. [27]. 4 mL of endoglucanase (0.43 FPU/mL) was used in the immobilization process. This amount was 1% (w·w^−1^) of enzyme (calculated as protein in the enzyme solution) based on powder support. The mixture was maintained for 24 hours under bland agitation at 25°C in 250 mL flask in shaker. After this step, the solid was separated from the liquid phase by filtering, and in both samples the protein concentration (Bradford), cellulolytic activity (FPU, Filter Paper Unit), and the yield of immobilization were determined. The best derivative was selected to be used in some derivatives of immobilization listed in Table 1. After the immobilization, the derivatives were packed at 5°C in acetate buffer (pH 5.6).

#### 2.3.2. Production of Drop and Sheet Derivatives

The production of derivatives follows the modified methodology of Albarghouthi et al. [28] and Tanriseven and Doğan [29].

*Drop*. Derivatives A, A′, F, and F′ were prepared by dripping the 3% (w·v^−1^) sodium alginate in 0.15 M CaCl_2_ (1 : 2 – v·v^−1^). Derivatives B, B′, G, and G′ were prepared by dripping in 3% (w·v^−1^) sodium alginate mixed with chitosan-acetic acid (1% (w·v^−1^) chitosan in 1% (w·v^−1^) acetic acid) in 0.15 M CaCl_2_ (1 : 2 - v·v^−1^). The derivatives were kept for 1 hour under gentle agitation at 25°C with or without activation with glutaraldehyde (Table 1).

*Sheet*. These derivatives were obtained by three procedures: (a) 3% (w·v^−1^) sodium alginate (derivatives C, C′, G, and G′); (b) 3% (w·v^−1^) sodium alginate mixed with chitosan-acetic acid (1% (w·v^−1^) chitosan in 1% (w·v^−1^) acetic acid) (derivatives D, D′, I, and I′); (c) 3% (w·v^−1^) sodium alginate mixed with chitosan-acetic acid (1% (w·v^−1^) chitosan in 1% (w·v^−1^) acetic acid) and 2% (w·v^−1^) of the* adsorbent* (E, E′, J, and J′). Solutions containing the supports were homogenized under constant mechanical stirring. The sheets were transferred to a polypropylene Petri dish (100 mm in diameter) in a layer of 2.5 mm thickness and dried in oven at 30°C for a period between 24 hours, reaching a thickness of 0.5 mm. Then 30 mL of 0.15 M CaCl_2_ was added on dried derivative which remained under mild agitation for 12 hours at 25°C. The derivatives were kept for 1 hour under gentle agitation at 25°C with or without activation with glutaraldehyde (Table 1).

### 2.4. Immobilization of 1,4-*β*-Endoglucanase by Entrapment in Alginate, Chitosan, and Latex

The 1,4-*β*-endoglucanase was immobilized by two types of procedure in solid (drop or sheet) support: (a) 1% (v·w^−1^) cellulase solution (calculated as protein based on mass of support) was mixed with the solution of sodium alginate and after 0.15 M CaCl_2_ solution was used for precipitation and formation of drops or sheets (A, A′, B, B′, C, C′, D, D′, E, and E′); (b) mixing sodium alginate (combined or not with other supports) with 0.15 M CaCl_2_ solution for the precipitation and formation of drops or sheets. After that, the drops or sheets were mixed with 1% (w·w^−1^) cellulase solution, calculated as protein based on the mass of support (F, F′, G, G′, H, H′, I, I′, J, and J′). Subsequently, part of derivatives was activated by the immersion in 0.5% glutaraldehyde water solution for 1 hour at 25°C [10]. After that, the derivatives were washed with deionized water (1000 mL) and packed in 5°C acetate buffer (pH 5.6) for use in tests of enzymatic activity. Cellulolytic activity (FPase, Filter Paper method) in derivatives was determined in one or more recycles (reuses).

The blend of alginate/chitosan/latex for endoglucanase immobilization was made in drops (3, 5, 7, and 10% m·m^−1^ latex) (Table 1, letters K, L, M, and N), with 3% (w·v^−1^) alginate solution and 1% (w·v^−1^) enzyme. The solution of chitosan-calcium was composed of 1% (w·v^−1^) chitosan, 1% (v·v^−1^) acetic acid, and 0.15 M CaCl_2_, and the derivatives were kept for 24 hours under bland agitation at 25°C in 250 mL flask in shaker. After this process, the derivatives were washed with deionized water (1000 mL) and packed in 5°C in acetate buffer (pH 5.6) for use in tests of the cellulolytic activity. The enzymatic stability was determined for successive recycles.

### 2.5. Enzyme Immobilization in Drops and Sheets

The 1,4-*β*-endoglucanase was also immobilized in two types of solid supports: drops in oval shape (Table 1, letters A, A′; B, B′; F, F′; G, G′) and sheets (Table 1, letters C, C′; D, D′; E, E′; H, H′; I, I′). The drops A, A′ and F, F′ were prepared by dripping 3% (w·v^−1^) sodium alginate on 0.15 M CaCl_2_ solution (1 : 2, v·v^−1^). Derivatives B, B′ and G, G′ were prepared by dripping 3% (w·v^−1^) sodium alginate mixed with 1% (w·v^−1^) chitosan in 1% (w·v^−1^) acetic acid with addition of 0.15 M CaCl_2_ (1 : 2 - v·v^−1^). In both derivatives the drops were kept for 24 hours under bland agitation at 25°C, with or without activation with 0.5% glutaraldehyde as previously described (Table 1). The drops presented a diameter of 5-6 mm.

**Figure 1:**
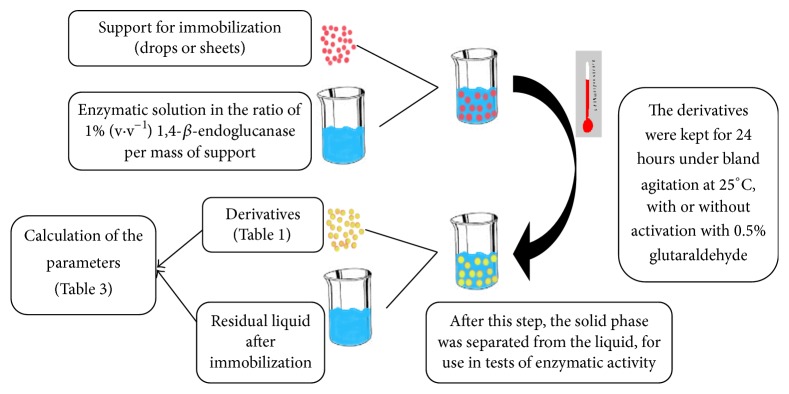
Flowchart of the enzyme immobilization process.

The sheets were obtained by three procedures: (a) solution of sodium alginate pure (derivative C, C′; G, G′), (b) mixture of the solution of alginate and chitosan (1 : 1) (derivatives D, D′; I, I′), and (c) sodium alginate solution with addition of 2% (w·v^−1^) of the best support obtained in the preliminary tests with activated carbon, zeolite, ion exchange resin, or polystyrene, derivatives E, E′; J, J′. Solutions containing the supports were homogenized under constant mechanical stirring. After that, these sheets were transferred to a polypropylene Petri dish (100 mm in diameter) in a layer of 2.5 mm thickness and dried in oven at 25°C for 24 hours, reaching a thickness of 0.5 mm. Then, 30 mL of 0.15 M CaCl_2_ was added on dried sheet which remained under bland agitation for 24 hours at 25°C, with or without activation with 0.5% glutaraldehyde as previously described (Table 1). The immobilization process is summarized in the flowchart (Figure 1).

### 2.6. Enzyme Immobilization in Drops with Latex

The drops (K, L, M, and N) were prepared by dropping the 3% (w·v^−1^) sodium alginate solution, latex (3, 5, and 10%, v·v^−1^), and 1% (w·v^−1^) enzyme in solution chitosan-calcium (1 : 2, v·v^−1^); the drops were kept for 24 hours under bland agitation at 25°C in 250 mL flask in shaker (Table 1). After this process, the derivatives were washed with deionized water (1000 mL) and packed in 5°C in acetate buffer (pH 5.6) for use in testing of the cellulolytic activity.

### 2.7. The Procedures of Derivatives with Latex in the enzyme Immobilization Process

The derivatives were prepared with latex in different concentrations as follows: (a) 5% latex (v·v^−1^) as a stabilizing agent, different chitosan concentrations (0.5 and 1% w·v^−1^ in 1% of acetic acid, v·v^−1^), and sodium alginate (w·v^−1^) (0 and 3%). 1% of enzyme solution (w·v^−1^) was added in 3% sodium alginate (w·v^−1^) and 5% latex (v·v^−1^); 3% sodium alginate (w·v^−1^) + 0.5% chitosan (w·v^−1^, 1% of acetic acid, v·v^−1^) + 5% latex (v·v^−1^); 3% sodium alginate (w·v^−1^) + 1% chitosan (w·v^−1^, 1% of acetic acid, v·v^−1^) + 5% latex (v·v^−1^); and 1% chitosan (w·v^−1^ in 1% of acetic acid, v·v^−1^) + 5% latex (v·v^−1^) (Table 1). In these derivatives, the drops were kept for 24 hours under bland agitation at 25°C in 250 mL flask in shaker. After this process, the derivatives were washed with deionized water (1000 mL) and packed at 5°C in acetate buffer (pH 5.6) for use in the tests of the cellulolytic activity.

### 2.8. Temperature

The temperatures of enzyme immobilization process (5, 10, 15, 25, 35, and 45°C) were performed with the best derivative in 24 hours in water bath (Tecnal, Piracicaba, SP, Brazil). Subsequently, the derivatives were washed with deionized water (1000 mL) and packed at 5°C in acetate buffer (pH 5.6) for use in the tests of the cellulolytic activity.

### 2.9. Analytical Procedure

#### 2.9.1. Determination of Total Cellulose Activity (Filter Paper Activity, FPase)

The enzymatic activity of free or immobilized endoglucanase was measured using the technique of FPase [30]. One unit of Filter Paper (FPU) activity was defined as the amount of enzyme releasing 1 *μ*mol of reducing sugar from Filter Paper per mL per min under the conditions described [31].

#### 2.9.2. Determination of Protein and Sugar Concentration

Protein was determined by Bradford method [32]. The determination of reducing sugars (glucose) liberated by hydrolysis of cellulose was carried out by 3,5-dinitrosalicylic acid (DNS) method under alkaline conditions [33].

### 2.10. Fourier Transform Infrared Spectroscopy (FTIR)

Fourier transform infrared spectroscopy (FTIR) was obtained to show the functional groups of drops (calcium alginate, latex, chitosan-calcium, and enzyme). The samples were measured directly by Attenuated Total Reflection (ATR) method, which is an efficient method for obtaining infrared information for the sample surface. The samples were characterized using a TENSOR 27 (Bruker, Germany) (500–6000 cm^−1^) with a resolution of 4 cm^−1^. Each reagent was analyzed separately and then together. As each group absorbs infrared radiation at a characteristic frequency, the inference of the presence of each group was possible comparing the radiation intensity versus frequency graph. Consequently, with this procedure, the determination of chemical interaction of materials was possible. The software Origin Pro 8® was used to make the statistical analysis of the data.

### 2.11. Compression Tests

The deformation and strength measures obtained by universal testing machine were analyzed to determine the stiffness of materials. Young's modulus or modulus of elasticity is obtained by the equation of the graph of stress and strain within the elastic limit of the reversible deformation; the function of this equation is called BiDoseResp (*y* = *A*1 + (*A*2 − *A*1)[*p*/1 + 10^(*LOGx*01 − *x*)*h*1^ + 1 − *p*/1 + 10^(*LOGx*02 − *x*)*h*2^]) [34, 35], which is the slope of the line [36]. Then this technique can measure the property of linear elastic solid materials. It measures the force (per unit area) that is needed to stretch (or compress) a material sample. A constant Young's modulus applies only to linear elastic materials. The material whose Young's modulus is very high can be approximated as rigid [36, 37]. The compression tests were carried out in a Universal Testing EMIC DL 2000 fitted with 10 kgf load cell at a speed. The cross-head speed employed was 10 mm/min. At least a triplicate of the samples was tested, and the average and standard deviation were reported. Prior to the tests, the samples were conditioned at 25°C. The mechanical compression test was conducted to examine the resistance to degradation of the drops. In this step, tests were performed with three different compositions: 3% sodium alginate (w·v^−1^) + 1% chitosan (w·v^−1^, 1% of acetic acid, v·v^−1^); 3% sodium alginate (w·v^−1^) + 1% chitosan (w·v^−1^ in 1% of acetic acid, v·v^−1^) + 5% latex (v·v^−1^); 3% sodium alginate (w·v^−1^) + 1% chitosan (w·v^−1^ in 1% of acetic acid, v·v^−1^) + 10% latex (v·v^−1^).

### 2.12. Calculation of the Parameters of Immobilization

The calculation of the parameters of immobilization and immobilized protein was calculated according to Silva et al. [10].

*(i) Immobilized Protein Yield (IPY)*. It was calculated as percentage of immobilized protein based on difference of supplied protein (*P*_0_) and the protein remaining in residual liquid after immobilization (*P*_*f*_), divided by *P*_0_ according to

(1) $$\begin{array}{c} IPY\left(\;\%\;\right)=\left[\;\frac{\left(P_{0}-P_{f}\right)}{P_{0}}\;\right]\times 100. \end{array}$$

*(ii) Enzyme Immobilization Yield (IY)*. It was calculated as percentage of immobilized enzyme based on the difference of supplied enzyme (*U*_0_) and the one remaining in the liquid after the immobilization (*U*_*f*_) (both expressed in FPU) divided by *U*_0_ according to

(2) $$\begin{array}{c} IY\left(\;\%\;\right)=\left[\;\frac{\left(U_{0}-U_{f}\right)}{U_{0}}\;\right]\times 100. \end{array}$$

*(iii) Recovered Activity (RA)*. It was calculated as percentage of immobilized enzyme (expressed in FPU) in the support (*U*_support_) divided by the difference between *U*_0_ and *U*_*f*_, according to

(3) $$\begin{array}{c} RA\left(\;\%\;\right)=\left[\;\frac{\left(U_{support}\right)}{\left(U_{0}-U_{f}\right)}\;\right]\times 100. \end{array}$$

The RA can be considered more precise immobilization yield than IY since the first is based on the enzyme activity measured in the derivatives (immobilized enzymes). There is a loss of activity in the immobilized enzyme, or not all enzymes remain active in the derivative.

*(iv) Lost Activity (LA)*. Since *U*_*f*_ can be recovered in another immobilization, LA represents the percentage of the lost enzymes in the total immobilization process, due to the difference of *U*_0_ and the sum of *U*_support_ and *U*_*f*_, according to

(4) $$\begin{array}{c} LA\left(\;\%\;\right)=\left[\;\frac{\left(U_{0}-\left(U_{support}+U_{f}\right)\right)}{U_{0}}\;\right]\times 100. \end{array}$$

*(v) FPU Yield (%)*. The increase in the FPU activity: this parameter was calculated by the sum of FPU in all recycled derivatives in enzymatic hydrolysis of Filter Paper (Σ⁡FPU), divided by *U*_0_, according to

(5) $$\begin{array}{c} FPU\;\;Yield=\left[\;\frac{\left(\Sigma FPU\right)}{\left(U_{0}\right)}\;\right], \end{array}$$

expressed in number of times.

*(vi) Net FPU Yield*. It is the net yield in FPU from Σ⁡FPU of the all recycled derivatives in enzymatic hydrolysis of Filter Paper in relation to the amount of supplied enzyme without *U*_*f*_. This parameter was calculated according to

(6) $$\begin{array}{c} Net\;\;FPU\;\;Yield=\frac{\Sigma FPU}{\left(U_{0}-U_{f}\right)}, \end{array}$$

expressed in number of times.

### 2.13. Statistical Treatment

Tests for enzymatic activity were conducted in triplicate, and data was submitted for analysis of variance (ANOVA), and the means were compared by Tukey test, using the program GraphPad Instat, Version 3.05 (Rutgers University). Curves and graphs were made in OriginLab software, Version 9.1, and Excel 2010. The treatments were analyzed statistically and considered significant at *p* < 0.05.

## 3. Results

### 3.1. Characterization of Commercial 1,4-*β*-Endoglucanase

The commercial 1,4-*β*-endoglucanase (ROHAMENT, CL) was analyzed by the total cellulase (Filter Paper activity, FPase) and total protein concentration (Bradford). In this way, the volumetric activity (FPU/mL) and specific activity (FPU/g protein) were determined in 3, 5, 12, and 20% (v·v^−1^) cellulase solution in acetate buffer at 40, 50, and 60°C (Table 2). The highest values of cellulase activity were obtained at 50°C with 20% (v·v^−1^) enzyme solution (0.67 ± 0.00 FPU/mL), although with a lower specific activity (72.94 ± 0.09 FPU/g protein) when compared to 3% (v·v^−1^) enzyme solution (0.34 ± 0.00 FPU/mL and 245.95 ± 0.15 FPU/g protein). Despite these higher results at 50°C, there were no statistical differences between 50 and 60°C (*p* > 0.05), but a significant difference at 40°C (*p* < 0.05). There was a decrease of more than 30% in activity in 20% (v·v^−1^) enzyme solution when comparing 40°C and 50°C (0.45 ± 0.02 and 0.67 ± 0.05, resp.). Therefore, the increase of the activity did not follow the same proportion of the increase of enzyme concentration, probably due to the limitation of the substrate concentration for the excess of enzyme.

### 3.2. Immobilization of 1,4-*β*-Endoglucanase on Activated Carbon, Zeolite, Ion Exchange Resin, and Polystyrene

Preliminary tests of the cellulase immobilization on activated carbon, zeolite, ion exchange resin, and polystyrene supports were performed for the selection of a promising solid support (Table 3). The highest and significant (*p* < 0.05) enzyme activity was obtained with the use of ion exchange resin (0.32 ± 0.02 FPU/g support) representing almost double. Therefore, this support was used in the derivatives of the sheets E, E′; J, J′ according to Table 1.

Despite zeolite and polystyrene showing a better yield of enzyme immobilization (IY) (83.3% and 68.8%, resp.), their Recovered Activity (RA) was, respectively, only 13.4% and 20%, reflecting the higher Lost Activity (LA) in FPU per gram of support. The best adsorption of endoglucanase was on the ion exchange resin. Even with this last support showing that IY of 33.34% reached higher RA (81.6%) due to lower LA (6.14%), reflecting positively in the immobilized activity. The activated carbon showed no immobilization yields (Table 3).

### 3.3. Immobilization of 1,4-*β*-Endoglucanase by Pure and Mixed Alginate

These derivatives were made by adding the enzyme during the preparation of the derivatives before entrapment with CaCl_2_. Table 3 shows the results with the drops (A, A′, B, B′, K, L, and M) and sheets (C, C′, D, D′, E, and E′).

The drops of the derivatives B and B′ (Table 3) showed, respectively, the highest FPase activity (0.15 and 0.17 FPU/g of support) and IY (56.1 and 55.4%), although with a great loss of activity (LA, 42.1% and 39.6%, resp.). The content of the immobilized protein (IP) and Immobilized Protein Yield (IPY) were almost the same (IP, 12 mg protein/g support and IPY, 86%) in all derivatives (A, A′, B, and B′).

The sheet D showed higher active enzyme (0.12 FPU/g of support) than the sheets C, C′, D′, E, and E′ and higher RA (54.1%), lower LA (10.3%), and higher IY (22.4%) when compared to the other derivatives. The content of the immobilized protein (IP) and protein yield (IPY) in derivative E′ were better (IP 11.2% and IPY 93.8%) when compared to the others (Table 3).

In the derivatives of drops immobilization with the addition of latex on supports of alginate and chitosan, IPY was gradually increasing to 85, 98, and 99%, respectively, according to the increase of latex concentration (3, 5, and 10% latex) in immobilization derivatives (Table 3). This increase was also followed by the increase of cellulose activity from 0.018 to 0.084 FPU/g support. However, in the drops of the derivative B with only alginate and chitosan (without latex) the immobilized active enzyme was higher (0.153 FPU/g) but with a lesser RA (25%) and higher LA (42.1%) (Table 3). The latex makes the adsorption of the enzyme on support difficult. This hypothesis should be considered since in these immobilizations the total protein immobilized in the support (IP) was very low when compared with the other derivatives without latex. For example, in the drops of the derivatives B and B′ with only alginate and chitosan (without latex), the IP were 12 mg/g of support for each derivative, while only 1.9–2.1 mg/g of support was obtained for the derivatives with latex (derivatives K, L, and M) (Table 3). One possibility of explaining this problem was the presence of lysozyme/chitinase activity from* Hevea brasiliensis* latex [38] which could change the chemical structure of alginate and chitosan on some level.

### 3.4. Enzyme Immobilization by Immersion of Solid Supports in 1,4-*β*-Endoglucanase Solution

The results of the immobilization process by immersion of solid supports (drops or sheets) in enzyme solution are shown in Table 4 for the drops (F, F′, G, and G′) and sheets (H, H′, I, I′; and J, J′, sheets).

The drops of the derivatives F and F′ (Table 3) showed higher FPase activity (0.13 and 0.093 FPU/g of support) and although in both derivatives similar results were obtained, their IY showed a great difference. The derivative F′ showed a better IY (51.5%) than F (15.4%). However, the Recovered Activity (RA) of F′ was lower than the derivative F (RA 16% and 76%, resp.). This last derivative showed a low Lost Activity (LA, 3.7%), and consequently the cellulolytic activity was higher when compared to the derivative treated with glutaraldehyde (F′), in which the Lost Activity (LA) was 43.3%. The derivatives G and G′ showed IPY of 48.6% and 52.4% respectively, although they had lower IY (13.4 and 13.5%) and RA (51.4 and 55.7%) (Table 3).

Among the sheets (Table 3) prepared by enzyme immersion, higher protein immobilization was verified in derivative I′ (IP 4.5 mg/g and IPY 45.8%). However, the derivatives with added enzyme during the preparation of support were superior, since the derivative C′ showed IP of 9.6 mg/g and IPY of 97.5%. In sheets H and H′ higher cellulolytic activity (0.105 and 0.093 FPU/g support) than the others using enzyme immersion was verified. These sheets demonstrated an improvement in IY, but the presence of glutaraldehyde (derivative H′) caused a decrease in the RA (10.74%) if compared with RA in derivative H (15%). The derivatives I and I′ showed, respectively, higher IY (55.4% and 46.1%), but also higher LA (53.5% and 42.4%).

### 3.5. Effect of Temperature on Immobilization of 1,4-*β*-Endoglucanase

In the test of the temperature of immobilization derivative B′ was used since with this derivative was obtained the best results of FPU per gram of derivative (Table 4). The parameter IPY was similar for all temperatures, but the FPU/g was significantly higher (0.172 FPU/g of support) at 25°C than at other temperatures (*p* < 0.05). Similarly, the RA was the highest (28.5%) and the LA was the lowest value (39.6%). Therefore, these results indicated 25°C as more efficient in the immobilization process.

### 3.6. Immobilization of Endoglucanase in Alginate, Chitosan, and Latex

The immobilization of endoglucanase in hybrid support with alginate, chitosan, and latex was evaluated in two concentrations of chitosan (0.5 and 1%), with and without sodium alginate (Table 3).

The Immobilized Protein Yield (IPY) showed that the highest the concentration of chitosan, the greater this parameter: 3% sodium alginate (w·v^−1^) + 5% latex (v·v^−1^) (IPY 33.5%); 3% sodium alginate (w·v^−1^) + 0.5% chitosan (w·v^−1^, 1% of acetic acid, v·v^−1^) + 5% latex (v·v^−1^) (IPY 71.5%); 3% sodium alginate (w·v^−1^) + 1% chitosan (w·v^−1^, 1% of acetic acid, v·v^−1^) + 5% latex (v·v^−1^) (IPY 98.4%). For the derivative with only chitosan, no reliable results were observed, probably because an unstable drop was obtained.

The derivative prepared with only alginate showed the highest RA (100%), but when comparing the amount of FPU per gram of support (0.0025 FPU/g of support) this value was 69 times lower than the derivative B′ (0.172 FPU/g of support) (Table 3). The derivatives using 0.5% and 1% chitosan showed better results when compared to the drops without chitosan, respectively, 0.026 and 0.066 FPU/g of support (*p* < 0.05). Therefore, based on these results the synergism between chitosan and alginate requires a better and more effective enzyme immobilization with higher yields, as already shown at derivative B′. However, if the resistance and flexibility were also considered, alginate, chitosan, and latex are more interesting, according to the following tests.

### 3.7. Compression Tests

**Figure 2:**
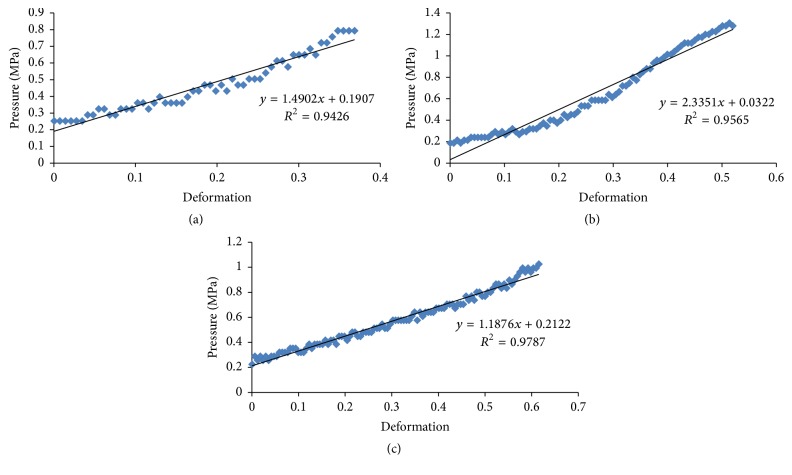
Different supports in behavior under compressive force. Supports are composed of (a) 3% sodium alginate (w·v^−1^) + 1% chitosan (w·v^−1^ - 1% of acetic acid - v·v^−1^); (b) 3% sodium alginate (w·v^−1^) + 1% chitosan (w·v^−1^ - 1% of acetic acid - v·v^−1^) + 5% latex (v·v^−1^) in behavior under compressive force; (c) 3% sodium alginate (w·v^−1^) + 1% chitosan (w·v^−1^ - 1% of acetic acid - v·v^−1^) + 10% latex (v·v^−1^).

The addition of latex into samples influenced the mechanical behavior. The latex became stiffer and resistant with a greater plastic deformation (more ductile), leading to higher interfacial interactions, acting as reinforcement or creating cross-linking [39]. The test of mechanical compression in three different compositions of latex in the drops was performed to determine their resistance to degradation: (a) 3% sodium alginate (w·v^−1^) + 1% chitosan (w·v^−1^, 1% of acetic acid, v·v^−1^); (b) 3% sodium alginate (w·v^−1^) + 1% chitosan (w·v^−1^, 1% of acetic acid, v·v^−1^) + 5% latex (v·v^−1^); (c) 3% sodium alginate (w·v^−1^) + 1% chitosan (w·v^−1^, 1% of acetic acid, v·v^−1^) + 10% latex (v·v^−1^). Therefore, the effect of latex concentration in the mechanical strength of these drops was evaluated, taking into account the fact that the more sturdy support for the immobilization of the enzyme suffers less degradation.  Figure 2(a) refers to the behavior of support alginate and chitosan in the test of compression, with the *x*-axis being the characteristic compressive strength of the material and the *y*-axis being the deformation (*y* = 1.4902*x* + 0.1907, *R*^2^ = 0.9426). In Figure 2(b) (*y* = 2.3351*x* + 0.0322, *R*^2^ = 0.9565) and Figure 2(c) (*y* = 1.1876*x* + 0.2122, *R*^2^ = 0.9787) the compression of the drops was analyzed according to the presence of latex (5 and 10%, v·v^−1^, resp.). The drops prepared with 3% (w·v^−1^) sodium alginate and 1% chitosan (without latex) showed the mean of the elasticity modulus 1.787 ± 1.033 MPa. However, the drops with 5 or 10% of latex (v·v^−1^) showed higher elasticity modulus (mean of 2.310 ± 0.160 Pa and 2.500 ± 1.140 MPa, resp.) than the drops without latex. Therefore, the addition of 5 and 10% latex in the samples influenced the mechanical behavior, increasing this parameter by 1.3 and 1.4 times, respectively.

### 3.8. FTIR Analysis of Endoglucanase Immobilized in Drops of Alginate, Chitosan, and Latex

**Figure 3:**
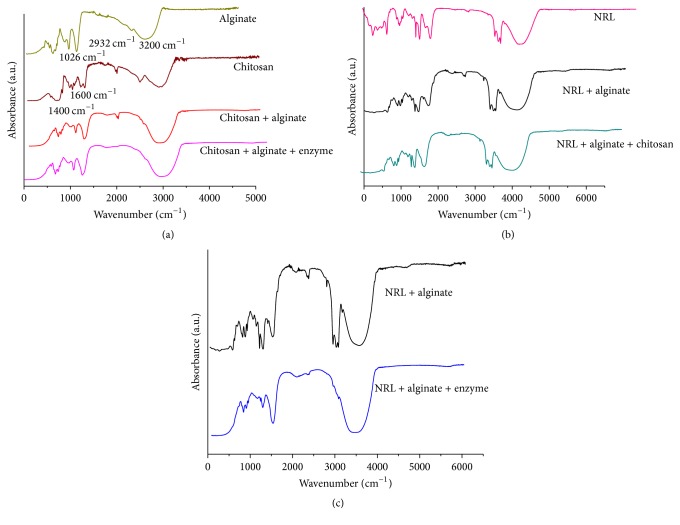
Infrared spectroscopy: (a) alginate, chitosan, blend, and enzyme loaded blend; (b) natural latex: NRL, NRL + alginate, and NRL + alginate + chitosan; (c) NRL + alginate and NRL + alginate + enzyme.

Each functional group of chemical compounds absorbs a characteristic frequency in the infrared region [40]. The infrared spectrogram can be used to characterize the functional groups of an unknown material. Spectra were obtained from the latex, sodium alginate, and chitosan and mixtures of these materials with each other and with the commercial enzyme used in this study.  Figure 3 shows the infrared spectra by Fourier transform, using module of “Attenuated Total Reflectance” (ATR) in the region 500–5000 cm^−1^.

Figure 3 showed significant absorption spectra for the materials, blends, and enzyme; the main bands are 1060 cm^−1^, 1090 cm^−1^, between 1070 and 1100 cm^−1^, between 1400 and 1500 cm^−1^, 1600 cm^−1^, 2600 cm^−1^, 3000 cm^−1^, 3200 cm^−1^, and 3400 cm^−1^. The chitosan spectrum (Figure 3) had two distortions between 1600 cm^−1^ and 3400 cm^−1^ related to a primary amine (NH_2_) obtained by deacetylation of chitin, a band near 3000 cm^−1^ (C-H bonds, organic compound) and 1400 cm^−1^ (alkyl groups and carboxylate O-C=O). Other bands were presented in 1060 cm^−1^ (C-O stretch vibrational primary alcohol); 1090 cm^−1^ (vibrational stretch ether group); and 1070–1100 cm^−1^ (aliphatic amines) [41].

The FTIR spectrum of sodium alginate is presented in Figure 3. Initially, the absorption peak observed at 3200–3500 cm^−1^ corresponds to stretching of hydroxyl groups, 2936 cm^−1^ is due to C-H stretching, and 1026 cm^−1^ is due to C-O-C stretching [42].

The interaction of sodium alginate with calcium chloride in mixtures causes a small change in the band near 1600 cm^−1^. Due to the connection and mixture with alginate the peaks related to the calcium chloride disappeared indicating the connection. The chitosan showed a band in 2000 cm^−1^ and 2600 cm^−1^, and vibration around 3200–3300 cm^−1^, and in the region between 1000 and 1700 cm^−1^. The IR of the blend chitosan/alginate there is bands in 2000 cm^−1^ and 2600 cm^−1^ and vibration between 1000 and 1700 cm^−1^. However, when the enzyme was mixed with this blend the bands in 2000 cm^−1^ and 2600 cm^−1^ were absent, indicating that there was a chemical interaction between enzyme and this blend (Figure 3).

The latex was employed in this study due to some interesting characteristics such as easy manipulation, low cost, and high mechanical resistance. The FTIR spectra of polymers blends (latex + chitosan, latex + alginate, and latex + chitosan + alginate) were analyzed. The pure latex spectrum (Figure 3(b)) had significant absorption near 2800 cm^−1^, which equals the bonds C-H present in abundance in the material. Another slight distortion that can be seen is 3200–3500 cm^−1^, indicating the presence of hydroxyl group. The band of absorption near 1400 to 1500 cm^−1^ indicated the presence of N-O and links that may be related to the ammonia present in the latex mixture to keep it liquid. In summary, FTIR spectral data confirmed the chemical stability of natural latex in alginate and chitosan blends [17].

However, Figure 3(c) presents FTIR spectra of blend (latex + alginate) before and after enzyme encapsulation. The most pronounced effect is a decrease of band absorbance at 2852–2925 cm^−1^ and 2961 cm^−1^ corresponding to CH_2_ symmetric and CH_3_ asymmetric stretching vibrations. It indicates the interaction between molecular chain of enzyme and this blend.

### 3.9. Recycle of Derivatives in Enzymatic Reaction of the Paper Filter as Substrate

**Figure 4:**
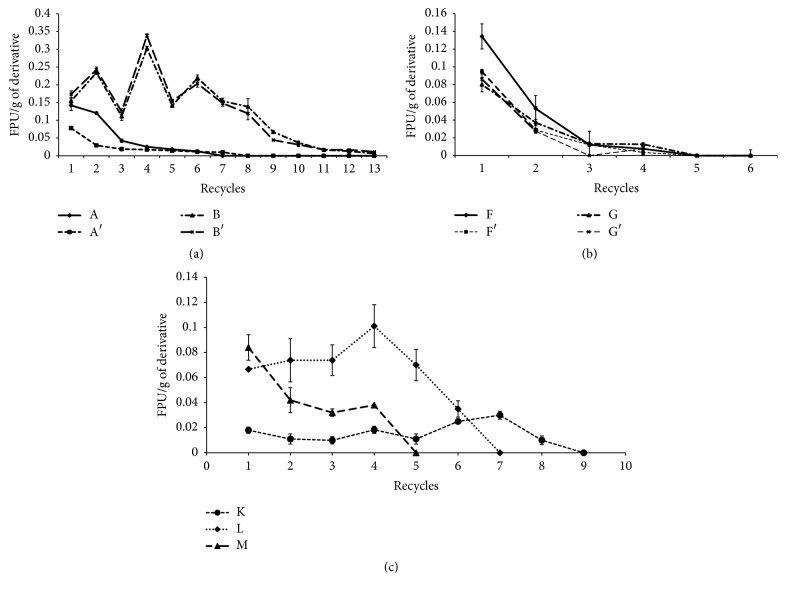
Recycle of the cellulases in drops on reactions with paper filter: (a) enzyme immobilized during the drop in production; (b) enzyme immobilized after of the drop was produced; (c) enzyme immobilized during the production of drop including latex (3, 5, and 10%). Derivatives are described in Table 1.

**Figure 5:**
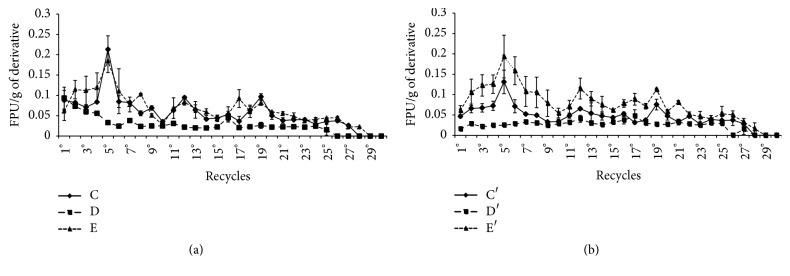
Recycle of cellulases on sheets in reactions with Filter Paper: (a) derivatives prepared with the enzyme adsorbed during preparation of sheets without glutaraldehyde; (b) derivatives treated with glutaraldehyde. Derivatives are described in Table 1.

The recycles of the drops (Figure 4) and sheets (Figure 5) that reacted with Filter Paper in water solution are presented. The recycles of all derivatives were quantified by the maximum number of recycles and the sum of FPU/g of support (Table 5). Derivatives A, A′, B, and B′ achieved greater stability over successive recycles of cellulolytic activity (Figure 4(a)). The drops achieved up to 13 recycles of immobilized enzyme in the derivatives B and B′, while in the derivatives F, F′, G, and G′ the cellulolytic activity was missed in the 5th recycle (Figure 4(a)). In the derivative B better results of its reuses was verified, and the total activity summed over 13 cycles was 1.59 FPU/g of support, while only 1.01 FPU/g of derivative B was obtained in the immobilized enzyme recycle. Therefore, there was an FPU Yield of 1.57 times higher in the cellulolytic activity than the total enzyme supplied for this immobilization method. However, if the Recovered FPU in liquid after immobilization was considered, the Net FPU Yield was 2.81 times (Table 6).

The stability of cellulolytic sheets was also evaluated by their recycles in reactions with paper filter as substrate and determination of FPase. The sheets (Figures 5(a) and 5(b)) showed greater stability in successive recycles of enzyme reaction. The sheets E and E′ were more efficient than the others since 28 reutilizations of derivatives were performed. The sheets H, H′, I, I′, J, and J′ showed a low stability since their recycles showed no activity after the 6th recycle. The sheets treated with glutaraldehyde have proved to be superior. The sheet E showed 1.95 FPU/g of support considering the total FPase activity summed in 28 cycles, but E′ reached 2.30 FPU/g of support, while only 1.4 FPU/g of derivatives E or E′ were supplied for the enzyme immobilization, or FPU Yield in sheet E was 1.64 times higher than the spent FPU (*U*_0_) (Table 6). In addition, the net balance between the enzyme spent for the immobilization and the sum of enzyme obtained with the reuse in sheet E′ (6.9 total FPU) means an increase of 5.3 times. These results demonstrate a great economy in the use of this enzyme by this method of enzyme immobilization.

The drops K with 3% latex (v·v^−1^) reached a higher stability during successive recycles of cellulolytic activity. The average of total activity was 0.17 FPU/g derivative, obtained by the sum of residual activities in 8 recycles (Figure 4(c)). The drops L with 5% latex (v·v^−1^) showed absence of cellulolytic activity in the 6th cycle, but the average of total activity was 0.42 FPU/g of support by the sum of residual activities in 5th recycle. An average of 0.19 FPU/g of support was obtained in the drops M with 10% latex (v·v^−1^) by the sum of 4 recycles of the immobilized enzyme (Figure 4(c)).

## 4. Discussion

Some aspects of endoglucanase immobilization process were studied, like shape (sheet or drop), the type of polymer used in support, the use of glutaraldehyde as cross-linking agent, and the step where the enzyme is added to produce the derivative. The evaluation of the best immobilization procedures was based on four main aspects: (a) cellulolytic activities effectively incorporated in the derivatives (FPU/g support), or the Recovered Activity (RA) which demonstrates the efficiency of the immobilization process, (b) the interaction of the latex in immobilization and stability of the supports, (c) the enzyme recycles, showing the stability of the chemical interaction of enzyme and solid support, and (d) the increase in the FPase activity in recycled derivatives, and its results in the economy of spending enzyme.

### 4.1. Immobilization on Activated Carbon, Zeolite, Ion Exchange Resin, and Polystyrene

Despite zeolite and polystyrene showing a better yield of enzyme immobilization, their Recovered Activity was low, probably due to the type of chemical interaction between the substrate and the enzyme, which was only through an adsorption. This type of immobilization is not selective and the electrostatic interaction can occur anywhere in the enzyme leading to inactivation of the catalytic site. Another consequence is the weak bonds between the carrier and the enzyme, contributing to the enzyme release during the reaction [43].

### 4.2. Effect of Chitosan and Glutaraldehyde in the Drops of Immobilized Endoglucanase

The presence of chitosan with alginate in drops B and B′ was probably responsible for the superior adsorption of the supplied enzyme compared to the derivatives A and A′, even with the great losses in residual liquid after immobilization (*U*_*f*_). The drop B′ was superior to B probably due to the presence of glutaraldehyde increasing positive groups (NH_2_) in combination with chitosan. In fact, there is a cross-linking reaction and the formation of a Schiff base (imine) by one glutaraldehyde molecule with one amino group, enhancing its aldol condensation with other glutaraldehyde molecules. The final cross-linked structure would be a linear aldol-condensed oligomer of glutaraldehyde, with several Schiff base linkages branching off [44]. The derivatives of the hybrid gels presented higher value of enzyme Immobilization Yield (IY), probably due to the presence of reactive free amine groups present in the chitosan and a greater affinity for proteins (Monteiro, Junior, 1999).

The addition of enzyme during the preparation of the drops probably makes the contact of cellulase and chitosan easier, and this reaction was probably responsible for the increase of IY. The RA (96%) in the drop A was superior than drops B and B′ (25% and 28.5%). If the catalytic site of the enzyme reacts with the amino groups of chitosan during the enzyme immobilization, these reactions could lead to the inactivation of the enzyme. This hypothesis is probably responsible for the increase of LA in the derivatives with chitosan, since LA was 42% in derivative B and 39.6% in derivative B′, comparing with only 6.47% in derivative A′ and only 0.53% in derivative A (Table 3).

### 4.3. Effect of Glutaraldehyde in the Sheets of Immobilized Endoglucanase

The enzyme Immobilization Yield (IY, 17.8%) and Recovered Activity (RA, 8.8%) in sheet D′ were lower than sheet D due to the glutaraldehyde (Table 3). In the derivatives C and C′ (alginate), higher values of IY, respectively, 36.3 and 53.5%, were observed. However, in these derivatives, there was a low RA (12.6 and 6.78%) and cellulolytic activity (0.063 and 0.05 FPU/g support) due to higher Lost Activity (LA, 31.8 and 49.9%). Therefore, the glutaraldehyde increased IY but decreased RA. This fact probably occurred due to the denaturant action caused by this agent on the enzyme indicating the sensitivity of endoglucanase to glutaraldehyde [45, 46]. Several enzymes have different behaviors in the presence of glutaraldehyde [45]. Spagna et al. [47] confirmed the degrading action of glutaraldehyde acting as enzyme inhibitor, inducing a total or partial loss of enzyme activity. A reticulation of xylanases and cellulases in drops of chitin-chitosan with glutaraldehyde was evaluated in concentrations from 0.125% to 1.5% for 0.5 hours. The most effective glutaraldehyde concentration was 0.125% and higher values prompted reduced activity [24]. This result confirms the inhibitory effect of glutaraldehyde verified in the present work.

In other derivatives (D′, E′, and I′) of enzyme immobilization in sheets with glutaraldehyde, a low IY was also verified. However, these results contrast with those obtained with the drop A′; therefore the drops are less sensitive to glutaraldehyde than sheets. The immobilization of cellulases in alginate sheets presented in this word is unprecedented, with no comparative studies in the literature.

### 4.4. Effect of Temperature in Immobilization of Cellulase

The decrease in the Immobilization Yield (IY) studied in extreme temperatures can be related to the physical characteristics of the reactants which make up the derivative. Thus, alginate viscosity variations are related to the temperature at which they are subjected, where every 5.6°C increase led to a reduction of 12% of viscosity [48]. In the same way the temperature decrease leads to an increase in viscosity of alginate. Therefore, the physical change that occurs in the alginate with the temperature variation could be related to the low efficiency of enzyme immobilization, since the glycosidic bonds were broken [48]. The physical properties of chitosan are also modified in different temperatures (Zanira-Mora et al., 2014), and the temperature changes the binding properties of the substrate with the enzyme component [49].

### 4.5. Immobilization Using Hydrogels

Chitosan was always necessary for effective immobilization, probably due to the presence of reactive free amine groups and this characteristic justifies a higher affinity for proteins (Monteiro-Junior and Airoldi, 1999) [10].

The interaction between the blend of chitosan and alginate is important in the formation of a hybrid gel. The biochemical constitution of chitosan was compounded by a deacetylated biopolymer (N-acetyl-D-glucosamine) which leads to a high number of reactive free amine groups. These charges are responsible for higher affinity for proteins [50]. However, the high amount of reactive free amine groups also gives higher solubility to chitosan [51]. The blend produced by these two compounds shows higher physical stability of the support, allowing greater resistance after enzyme recycles.

Zhang et al. [52] encapsulated *α*-transglycosidase in supports of chitosan-alginate under ideal conditions. The results were similar to the enzyme in free form, but with greater stability of pH and temperature. In another study, Ramirez et al. [53] immobilized pectinase in alginate-chitosan and had 70% Protein Yield and 60% Recovered Activity, while maintaining catalytic capability around 50% even after 9 cycles of reuse. Saleem et al. [54] immobilized endoglucanase in polyacrylamide gel and achieved 53.4% Immobilization Yield (IY), near the value obtained in this study for immobilization in alginate-chitosan (42.63% and 48.08%). Furthermore, the enzyme activity remained even after five repeated cycles of applications. Silva et al. [10] working with immobilization of papain on chitin treated with PEI (polyethyleneimine) and chitosan cross-linked with TPP (tripolyphosphate) managed 6.07% and 15.7%, respectively, for yields of active immobilized enzyme. These results were lower than those obtained in the present work.

The activity FPU/g of derivatives produced with latex (drops K, L, and M) was approximately 1.93% lower than other values obtained by the immobilization derivatives without latex. In this case, the latex did not help in the increase of the Immobilization Yield (IY), even with the increase of Immobilized Protein Yield (IPY). The free amine groups of chitosan and cellulases may have been inhibited by the latex (Monteiro Jr, 1999), [10].

### 4.6. The Immersion of the Drops and Sheets in Enzyme Solution

The presence of chitosan in drops G and G′ was responsible for the major adsorption of the supplied enzyme, although a lower YI and RA were obtained when compared to the derivative F (Table 3). The drop G′ was superior to drop G probably due to the action of glutaraldehyde in the enzyme.

The immersion of sheets in enzyme solution disfavors the protein immobilization on the support. The IP and IPY in these derivatives were lower than the derivatives of the enzyme mixed during the production of sheets. This fact shows the importance of the preparation method of the derivatives. Sheets were less efficient than drops produced by enzyme immersion on support. The drop F (only alginate) showed 0.134 FPU/g support, RA of 76.01%, and only 3.7% LA, while sheet H showed only 0.105 FPU/g support, RA of 15%, and 24.2% LA.

### 4.7. Sheets × Drops of Immobilized Endoglucanase

The content of the immobilized protein (IP) in drops was better than in sheets (Table 3), but the sheet E′ showed higher IPY than the others probably due to the presence of ion exchange resin and cross-linking with glutaraldehyde. However, the cellulolytic activities in sheets (sheet H, 0.105 FPU/g, or sheet E, 0.085 FPU/g) were lower than in the best drops (drop B′, 0.172 FPU/g, drop B, 0.153 FPU/g). The comparison of RA and LA between sheet and drop of immobilized endoglucanase revealed a higher RA (76–96%) and lower LA (0.53–3.7%) in the drops (A and F), than the best sheets (D and H, RA = 15–54% and LA 10–24%). However, there is an important difference between these two processes. The process of sheet production had a final drying step (25°C for 24 hours) which did not occur with the process of drops production. This drying may compromise the catalytic site of the enzyme leading to an increase of LA and a decrease of RA. On the other hand, this evaluation is just for one cycle of reaction. The strength of these adsorptions must also be evaluated by the recycle of these derivatives to conclude which process is more advantageous.

### 4.8. Latex Blend: The Ability to Resist Deformation and Mechanical Compression Test

The high values of elasticity for only sodium alginate + chitosan (1.787 MPa) demonstrated that the use of calcium alginate in the hydrogel matrix formation provides an increase in the stiffness and compressive strength, according to de Moura et al. [36]. However, chitosan is important for deformation. The mechanical behavior of polymer blends with chitosan and collagen was studied by Tonhi and Plepis [55]. They observed a larger deformation suffered in these blends with higher amounts of chitosan. In the present work, the results obtained without latex were lower than 5% latex (2.31 MPa) or 10% latex (2.50 MPa). The presence of the latex increased this parameter compared to derivatives only with alginate and chitosan, demonstrating that the latex blends become more elastic and resistant to deformation. Fresh natural latex is composed of 33% of hydrocarbons as* cis*-1,4-polyisoprene, and this product undergoes processes to achieve up to 60% of rubber in the composition providing a wide elastic characteristic [37]. Simões et al. [56] assert that the natural latex has its own characteristics such as elasticity, plasticity, and wear resistance. Therefore, the addition of latex to immobilization increased their resistance to degradation.

### 4.9. FTIR Analysis in Different Materials

The bands at 1060 cm^−1^ are linked chemical bonds of the primary alcohol at 1090 cm^−1^ to vibrational stretch ether group, and at 1070–1100 cm^−1^ related to aliphatic amines (Torres et al., 2005). The interaction of sodium alginate with calcium chloride in the mixture caused a small change, which can be analyzed by the band near 1600 cm^−1^ [57]. The mixture of chitosan with alginate (Figure 3(a)) is found to change after complexation between both, where the bands represent the symmetrical and asymmetrical connections CO_2_^−^ [58]. Moreover, the bands in the region between 1700 and 1000 cm^−1^ characteristic of alginate and absent in chitosan, after this mixture still present, indicate an effective interaction between the polymers and the prevalence of alginate in the end bracket [58].

The mixture of chitosan + alginate (Figure 3(a)) has the bands representing the symmetrical and asymmetrical connections CO_2_^−^ change very slightly after complexation [58]. The spectrum of the mixtures also shows changes relative to the chitosan spectrum, with the bands relative to the primary amino groups (NH_2_) at about 3400 cm^−1^ (Figure 3(a)). The changes in the amino and carboxyl groups may indicate the bond between the compounds. There are bands in the region between 2600 and 3400 cm^−1^ relating to ammoniums groups in chitosan curve that is absent in the alginate curve and lost in this blend, indicating interactions among these carbohydrates. The band of 2600 cm^−1^ is only present in chitosan and the blend. Moreover, the bands in the region between 1700 and 1000 cm^−1^ in the alginate curve, still present and absent in complexing with chitosan, indicate an effective interaction between the polymers and the prevalence of alginate in the end bracket [41, 58].

The band at 2800 cm^−1^ is present in latex and the blend latex has more alginate. However, when the chitosan was mixed, this band was absent, indicating the interaction between chitosan and latex (Figures 3(a) and 3(b)). The vibration in 2800 cm^−1^ is characteristic of chitosan [17]. However, in the curves where there is the presence of cellulolytic enzyme, the disappearance of the bands of some other component was observed (Figure 3), such as ammonium groups of chitosan (around 3300 cm^−1^), carbon-hydrogen bonds of latex, and carboxyl groups of alginate. These changes may indicate that interactions actually occur between the enzyme and the support.

### 4.10. Recycle of Derivatives in Enzymatic Reaction with Filter Paper as Substrate

The derivatives prepared by immersion of supports in endoglucanase solution proved to be more unstable after their recycles on paper filter reaction. The number of recycles in drops or sheets was inferior to the method of inclusion enzyme during the preparation of derivatives, independently of the treatment with glutaraldehyde, type of support (pure or hybrid), or the shape (drop or sheet).

The presence of glutaraldehyde was not relevant either in the immobilization of drops or sheets, since the number of enzyme recycles was the same for all derivatives using the preparation method by immersion of supports in endoglucanase solution. In contrast, the number of recycles was influenced by the shape, since the sheets were recycled up to 28 times (Figure 5), while drops only up to 13 times (Figures 4(a) and 4(b)).

The type of the mixture of the supports was also relevant in the immobilization process, since drops of the blend alginate and chitosan showed higher activity. The drops B showed greater stability in recycles, with a total of 13 reuses, FPU Yield of 1.57 times, and Net PFU Yield of 2.81 times based on used enzyme. However, the sheet E′ showed the highest stability in 28 reuses of enzyme, PFU Yield of 1.64 times, and Net FPU Yield of 5.31 times proving to be the most efficient treatment saving more enzyme by the immobilization process (Figures 3 and 4). These results were better than those found in the literature. In a study using cellulases obtained from* Bacillus subtilis* TD6 immobilized in calcium alginate only 4 reuses of cellulases were obtained [59]. In another study with a *β*-1,3-glucanase from* Trichoderma harzianum* immobilized in calcium alginate up to 7 reuses of these enzymes were achieved without losing the enzymatic activity (El-Katatny, 2008). Zhang et al. [60] working with immobilization of lipase in alginate hydrogel beads maintained a high activity only in five cycles. Saleem et al. [54] showed only 6 times of enzyme reuse with immobilizing endoglucanase in polyacrylamide gel. Romo-Sánchez et al. [24] immobilized cellulases and xylanases in different polymers in two steps. In the first, the improvement of the adsorption of these enzymes in alginate-chitosan and chitin-chitosan was performed. In a second step, the reticulation (adsorption and cross-linking) was performed after improvement of the adsorption conditions for the chitin-chitosan support by adding 0.125% glutaraldehyde to the chitosan-enzyme system for 0.5 hours. In these conditions they obtained enzyme stability up to 19 cycles retaining 64% activity.

The drops of alginate, chitosan, and latex although demonstrating a high Immobilized Protein Yield (IPY) and moderate stability, did not overcome the performance of the derivative B (without latex), with 13 recycles and 1.59 FPU/g in the sum of all recycles, since, in the derivative using 3% latex (drop K) with 8 recycles, only 0.17 FPU/g of support was obtained. The drops of 3% latex (v·v^−1^) showed greater stability than 5 or 10% latex (v·v^−1^) (8, 6, and 5 recycles, resp.), but with a lower cellulolytic activity in each cycle, dropping more than 50% in the first recycle. Therefore, the presence of latex in the concentrations evaluated did not favor the immobilization of cellulases and its stability, probably due to the interaction of the enzyme with the support or the action of some carbohydrases presented in the natural latex [38]. The improvement of this technology is possible by the study of purity of cellulases, the ratio of enzyme and support, quality of the supports, pH, time of immobilization, and other functional agents. The cellulosic residues such as cardboard, paper, newspaper, and pretreated lignocellulosic residues could be used to produce fermentable and low cost sugars with wide application in the biotechnology industry, as well as reduction of waste and environmental damage.

The results obtained in the present work showed the immobilization of endocellulase and its recycle are technically possible, preferably using the enzyme added during the step of preparation of derivatives. The number of reuses of immobilized enzyme was relevant and this method must be considered to be applied on an industrial scale.

## Figures and Tables

**Table 1:** The derivatives of cellulase immobilization.

Type of supports	Shape	Names of derivatives
Calcium alginate +1% enzyme	Drop	A
Calcium alginate + 1% enzyme	Drop	A′ (glutaraldehyde^3^)
Calcium alginate + chitosan + 1% enzyme	Drop	B
Calcium alginate + chitosan + 1% enzyme	Drop	B′ (glutaraldehyde)
Calcium alginate + 1% enzyme	Sheet	C
Calcium alginate + 1% enzyme	Sheet	C′ (glutaraldehyde)
Calcium alginate + chitosan + 1% enzyme	Sheet	D
Calcium alginate + chitosan + 1% enzyme	Sheet	D′ (glutaraldehyde)
Calcium alginate + adsorbent^1^ + 1% enzyme	Sheet	E
Calcium alginate + adsorbent^1^ + 1% enzyme	Sheet	E′ (glutaraldehyde)
Calcium alginate (EAAP^2^)	Drop	F
Calcium alginate (EAAP)	Drop	F′ (glutaraldehyde)
Calcium alginate + chitosan (EAAP)	Drop	G
Calcium alginate + chitosan (EAAP)	Drop	G′ (glutaraldehyde)
Calcium alginate (EAAP)	Sheet	H
Calcium alginate (EAAP)	Sheet	H′ (glutaraldehyde)
Calcium alginate + chitosan (EAAP)	Sheet	I
Calcium alginate + chitosan (EAAP)	Sheet	I′ (glutaraldehyde)
Calcium alginate + adsorbent (EAAP)	Sheet	J
Calcium alginate + adsorbent (EAAP)	Sheet	J′ (glutaraldehyde)
Calcium alginate + 3% latex + chitosan-calcium + 1% enzyme	Drop	K
Calcium alginate + 5% latex + chitosan-calcium + 1% enzyme	Drop	L
Calcium alginate + 10% latex + chitosan-calcium + 1% enzyme	Drop	M

^1^The adsorbent was cation and anion exchanger resin (Amberlite, MB-20); ^2^EAAP: enzyme adsorbed after drop production; ^3^treatment with 0.5% glutaraldehyde for 1 hour.

**Table 2:** Cellulase activity of 1,4-*β*-endoglucanase (ROHAMENT, CL) in different concentrations and temperatures in pH 5.6.

Enzyme concen. (%) (v·v^−1^)	Protein concen. (g·mL^−1^·10^−4^)	40°C		50°C^2^		60°C^2^	
		FPU/mL	FPU/g	FPU/mL	FPU/g	FPU/mL	FPU/g
3	13.83 ± 1.15^a^	0.12 ± 0.01^a,a′^	90.75 ± 1.1^a,a′^	0.34 ± 0.05^a,b′^	245.95 ± 10.4^a,b′^	0.30 ± 0.04^a,b′^	236.95 ± 12.2^a,b′^
5	37.43 ± 3.01^b^	0.16 ± 0.00^b,a′^	44.93 ± 5.05^b,a′^	0.43 ± 0.03^b,b′^	117.28 ± 9.1^b,b′^	0.41 ± 0.02^b,b′^	108.28 ± 10.1^b,b′^
12	77.89 ± 09.05^c^	0.37 ± 0.03^c,a′^	48.76 ± 3.10^b,a′^	0.61 ± 0.05^c,b′^	78.50 ± 7.0^c,b′^	0.59 ± 0.03^c,b′^	69.50 ± 09.0^c,b′^
20	92.71 ± 12.03^d^	0.45 ± 0.02^d,a′^	49.21 ± 3.09^b,a′^	0.67 ± 0.05^c,b′^	72.94 ± 5.0^c,b′^	0.65 ± 0.05^c,b′^	71.0 ± 5.0^c,b′^

Different letters indicate that they are statistically different (*p* < 0.05), without lines indicate comparison between concentrations, and with lines between temperatures.

**Table 3:** Parameters of cellulase immobilized in different derivatives.

Derivatives	IP^1^	IPY^2^	FPU/g^3^	IY^5^ (%)	RA^6^ (%)	LA^7^ (%)
*Control-free enzyme (5%)*			0.438^4^	100	100	—
*Drops*						
A (calcium alginate)	11.9	85.6	0.141 ± 0.01^a^	13.4	96.0	0.53
A′ (calcium alginate + glut.^8^)	12.0	86.1	0.077 ± 0.02^b^	13.5	52.2	6.47
B (calcium alginate + chitosan)	12.0	86.3	0.153 ± 0.06^a^	56.1	25.0	42.1
B′ (calcium alginate + chitosan + glut.)	12.0	86.5	0.172 ± 0.01^a^	55.4	28.5	39.6
F (calcium alginate)	5.0	48.8	0.134 ± 0.03^a^	15.4	76.0	3.7
F′ (calcium alginate + glut.)	5.3	52.0	0.093 ± 0.02^a^	51.5	16.0	43.3
G (calcium alginate + chitosan)	4.9	48.6	0.080 ± 0.00^b^	13.5	51.4	6.6
G′ (calcium alginate + chitosan + glut.)	5.3	52.4	0.086 ± 0.01^b^	13.4	55.7	6.0
K (calcium alginate + chitosan + 3% latex)	1.9	84.9	0.018 ± 0.00^c^	49.37	44.8	27.2
L (calcium alginate + chitosan + 5% latex)	2.0	98.4	0.066 ± 0.01^b^	42.6	62.2	16.0
M (calcium alginate + chitosan + 10% latex)	2.1	99.5	0.084 ± 0.03^b^	45.8	73.0	12.3
*Sheets*						
C (calcium alginate)	9.3	94.7	0.063 ± 0.02^m^	36.3	12.6	31.8
C′ (calcium alginate + glut.)	9.6	97.5	0.050 ± 0.00^m^	53.5	6.78	49.9
D (calcium alginate + chitosan)	5.9	33.1	0.120 ± 0.03^n^	22.4	54.1	10.3
D′ (calcium alginate + chitosan + glut.)	5.9	33.1	0.015 ± 0.01^o^	17.8	8.77	16.2
E (calcium alginate + resin)	10.6	88.8	0.085 ± 0.01^m^	36.2	14.7	30.9
E′ (calcium alginate + resin + glut.)	11.2	93.8	0.049 ± 0.01^m^	30.8	10.0	27.7
H (calcium alginate)	2.7	27.9	0.105 ± 0.02^n^	28.5	15.0	24.2
H′ (calcium alginate + glut.)	3.2	32.4	0.093 ± 0.00^m^	35.3	10.7	31.5
I (calcium alginate + chitosan)	0.1	1.1	0.047 ± 0.00^m^	55.4	3.5	53.5
I′ (calcium alginate + chitosan + glut.)	4.5	45.8	0.089 ± 0.03^m^	46.1	7.9	42.4
J (calcium alginate + resin)	1.4	15.0	0.075 ± 0.00^m^	23.1	13.2	20.0
J′ (calcium alginate + resin + glut.)	1.6	16.0	0.091 ± 0.00^m^	24.6	15.2	20.8
*Other derivatives*						
Activated carbon	0.0	0.0	0.00 ± 0.00	0.0	0.0	100.0
Zeolite	0.46	4.6	0.13 ± 0.01^b^	83.3	13.4	72.1
Ion exchange resin	0.97	9.7	0.32 ± 0.02^c^	33.3	81.7	6.1
Polystyrene	2.26	22.7	0.16 ± 0.05^b^	68.8	20.0	55.0
3% calcium alginate + 5% latex	1.7	33.5	0.0025 ± 0.00^a^	35.0	100.0	—
3% calcium alginate + 0.5% chitosan + 5% latex	2.0	71.5	0.026 ± 0.00^b^	48.0	49.2	24.9
3% calcium alginate + 1% chitosan + 5% latex	2.0	98.4	0.066 ± 0.00^c^	42.6	62.2	16.0
1% chitosan + 5% latex^9^	0.0	0.0	0.0	0.0	0.0	100.0

^1^Amount of immobilized protein (mg protein/gram of support); ^2^Immobilized Protein Yield (%); ^3^Enzymatic activity (FPU) per gram of support; ^4^Enzymatic activity (FPU/mL) of enzyme solution (5%); ^5^Immobilization Yield (%); ^6^Recovered Activity (%); ^7^Lost Activity (%); ^8^Treatment with 0.5% glutaraldehyde (%); ^9^there was no solubilization of chitosan in latex. Obs. different letters indicate that they are statistically different (*p* < 0.05).

**Table 4:** Parameters of cellulase immobilized (derivative B′) in different temperatures.

Temperature of immobilization (°C)^1^	IP^2^	IPY^3^	FPU/g^4^	IY^5^ (%)	RA^6^ (%)	LA^7^ (%)
5	11.9	85.8	0.087 ± 0.01^a^	81.7	9.7	73.7
10	12.1	87.2	0.091 ± 0.03^a^	80.4	10.3	72.0
15	11.9	85.6	0.093 ± 0.05^a^	53.7	15.8	45.2
25	12.0	86.5	0.172 ± 0.01^b^	55.4	28.5	39.6
35	12.0	86.4	0.065 ± 0.10^c^	57.0	10.4	51.1
45	12.1	87.0	0.069 ± 0.08^c^	57.9	10.9	51.6

^1^Derivative B′ (alginate + chitosan + glut.); ^2^amount of immobilized protein (mg protein/gram of support); ^3^Immobilized Protein Yield (%); ^4^Activity FPU/g of support; ^5^Immobilization Yield (%); ^6^Recovered Activity (%); ^7^Lost Activity (%). Obs. different letters indicate that they are statistically different (*p* < 0.05).

**Table 5:** Number of recycles of celluloses immobilized in pure and hybrid derivatives on reaction with paper filter as substrate.

Derivat^1^	Glut.^2^	Recycle number				Sum of FPU in all recycles (FPU/g)			
		Enzyme added during derivative preparation		Enzyme immerged on support		Enzyme added during derivative preparation		Enzyme immerged on support	
		Sheet	Drop	Sheet	Drop	Sheet	Drop	Sheet	Drop
Calcium align.	No	27 (C)	6 (A)	4 (H)	4 (F)	1.72	0.36	0.25	0.20
	Yes	27 (C′)	7 (A′)	4 (H)	4 (F′)	1.37	0.17	0.25	0.13
Calcium align. + chitosan	No	25 (D)	13 (B)	1 (I)	4 (G)	0.81	1.59	0.05	0.14
	Yes	25 (D′)	13 (B′)	5 (I′)	4 (G′)	0.74	1.62	0.43	0.12
Calcium align. + resin	No	28 (E)	NP^c^	4 (J)	NP	1.95	NP	0.16	NP
	Yes	28 (E′)	NP	5 (J′)	NP	2.30	NP	0.24	NP
3% latex	No	NP	8 (K)	NP	NP	NP	0.17	NP	NP
5% latex	No	NP	6 (L)	NP	NP	NP	0.42	NP	NP
10% latex	No	NP	4 (M)	NP	NP	NP	0.19	NP	NP

^1^The total of endoglucanase supplied to prepare each derivative was A, A′, F, F′, G, G′ = 1.10 FPU/g support, B/B′, K/L/M – 1.01 FPU/g support; C/C′, E, E′ = 1.4 FPU/g support, D, D′ = 0.98 FPU/g support, G, G′ = 1.1 FPU/g support, H, H′ = 1.22 FPU/g support; I, I′ = 1.81 FPU/g support, J/J′  =  0.868 FPU/g support; ^2^Treated with 0.5% glutaraldehyde in 1 hour. ^c^Not performed.

**Table 6:** General overview of immobilization process in derivatives B and E′.

Item	Data	Drop B	Sheet E′
1	Number of recycles	13	28
2	Mass of derivative	15 g	3 g
3	Total enzyme supplied (*U*_0_)	15.15 total FPU	4.2 total FPU
4	Supplied Enzyme per gram	1.01 FPU	0.047 FPU
5	Activity in liquid after the immobilization (*U*_*f*_)	6.65 total FPU	2.90 total FPU
6	Total activity in support (*U*_support_)	2.12 FPU	0.14 FPU
7	Total Lost Activity	6.38 FPU	1.16 FPU
8	*U*_0_ − *U*_*f*_	8.5 total FPU	1.3 total FPU
9	Sum of FPU in all recycles	23.85 total FPU	6.90 total FPU
10	FPU Yield	1.57 times	1.64 times
11	Net FPU Yield	2.81 times	5.31 times

Item 4 = item 3/item 2; item 7 = item 3 − (item 6 + item 5); item 8 = item 3 − item 5; item 10 = item 9/item 3; item 11 = item 9/8.
